# Protective Immune Response against Bacillus anthracis Induced by Intranasal Introduction of a Recombinant Adenovirus Expressing the Protective Antigen Fused to the Fc-fragment of IgG2a

**Published:** 2014

**Authors:** D. N. Shcherbinin, I. B. Esmagambetov, A. N. Noskov, Yu. O. Selyaninov, I. L. Tutykhina, M. M. Shmarov, D. Yu. Logunov, B. S. Naroditskiy, A. L. Gintsburg

**Affiliations:** Gamaleya Research Institute for Epidemiology and Microbiology, Ministry of Public Health of the Russian Federation, Gamaleya Street 18, 123098, Moscow, Russia; National Research Institute for Veterinary Virology and Microbiology of Russia, Russian Academy of Agricultural Sciences, 601120, Pokrov, Vladimir region, Russia

**Keywords:** Bacillus anthracis, immunization, protective antigen, recombinant adenovirus

## Abstract

Anthrax is a particularly dangerous infectious disease that affects humans and livestock. It is characterized by intoxication, serosanguineous skin lesions, development of lymph nodes and internal organs, and may manifest itsself in either a cutaneous or septic form. The pathogenic agent is Bacillus anthracis, a grampositive, endospore-forming, rod-shaped aerobic bacterium. Efficacious vaccines that can rapidly induce a long-term immune response are required to prevent anthrax infection in humans. In this study, we designed three recombinant human adenovirus serotype-5-based vectors containing various modifications of the fourth domain of the B. anthracis protective antigen (PA). Three PA modifications were constructed: a secretable form (Ad-sPA), a non-secretable form (Ad-cPA), and a form with the protective antigen fused to the Fc fragment of immunoglobulin G2a (Ad-PA-Fc). All these forms exhibited protective properties against Bacillus anthracis. The highest level of protection was induced by the Ad-PA-Fc recombinant adenovirus. Our findings indicate that the introduction of the Fc antibody fragment into the protective antigen significantly improves the protective properties of the Ad-PA-Fc adenovirus against B. anthracis.

## INTRODUCTION

*Bacillus anthracis *is a gram-positive, endospore-forming, rod-shaped aerobic bacterium that causes a dangerous infectious disease that affects susceptible animals and humans. Human anthrax cases are reported every year in many countries. Anthrax spores penetrate the body and are absorbed by macrophages, which, in turn, migrate to the local lymph nodes [1]. Inside the macrophages, the spores evolve into a vegetative form, which causes progression of the generalized infection. Due to the *B. anthracis *pathogenicity, anthrax often becomes an acute, highly lethal disease, unless preventive and curative interventions are undertaken on time [2-5].

Even today, the problem of anthrax prevention remains important, because of the yearly sporadic disease outbreaks with lethal outcomes in humans [6, 7]. In Russia, a vaccine containing the acapsular strain STI-1 is used for anthrax prevention. However, the live spore vaccine STI-1 has a number of disadvantages, including the need for annual re-vaccinations, reactogenicity, and absence of a strong immunity against certain field isolates circulating in Russia [8-12]. The chemical vaccine used in the USA is not an ideal option, as it requires six-dose vaccination series over 18 months to develop a strong immunity, which causes allergization of the re-vaccinated organism. Taking this factor into account, the issue of further exploring g anthrax vaccines remains important both in the medical and veterinary practice: therefore, the search for means of specific anthrax prophylaxis continues.

One of the first attempts to use the adenoviral vector to immunize laboratory animals against *B. anthracis* was undertaken by a U.S. research group led by M.J. McConnell [13]. The researchers achieved expression of the secretable modification of the fourth domain of the protective antigen using the recombinant adenoviral vector and demonstrated that a single immunization of experimental animals with subsequent introduction of a lethal dose of the anthrax toxin provided 67% protection to Balb/c mice. These data indicated for the first time that adenoviral vectors carrying genes of the main protective antigen determinants could be successfully used for immunization against anthrax.

We generated recombinant adenoviruses capable of inducing a specific immune response against *B. anthracis*. The construct contained an insertion encoding a fusion protein consisting of the fourth domain of the protective antigen (PA) and the Fc-fragment of IgG2a. Two recombinant adenoviruses were genetically engineered as controls. The above-mentioned adenoviruses carried secretable and non-secretable modifications of the PA fourth domain. All three variants showed immunogenic and protective properties, inducing the synthesis of specific antibodies against *B. anthracis*. However, a recombinant adenovirus containing the insertion encoding the fusion protein (Ad-PA-Fc) demonstrated the highest level of protection in comparison with the controls Ad-sPA and Ad-cPA.

## EXPERIMENTAL

**Generation of recombinant adenoviruses**

The codons in the gene encoding the protective antigen were optimized by *in silico *analysis. The two most frequent amino acid triplets were used to modify the codons in the PA-Fc gene to be expressed in *Mus musculus* cells. The most frequent codons of *M. musculus* were defined in accordance with the following database: http://www.kazusa.or.jp/codon/. The modified nucleic acid sequence encoding the fourth domain of PA fused to the Fc-fragment of antibody was synthesized by ZAO Eurogen and delivered to us in the form of a pAtlas-PA-Fc plasmid.

The NotI- and HindIII sites of the PA-Fc fragment were cloned into the shuttle vector pShuttle-CMV in order to obtain the shuttle plasmid pShuttle-CMVPA- Fc. After that, the plasmids pShuttle-sPA and pShuttle-CMV-cPA were retrieved via restriction and subsequent ligation. pShuttle-CMV-sPA was obtained via the splitting of pShuttle-CMV-PA-Fc with XhoI restriction endonuclease, followed by ligation of the sticky ends. The corresponding C-end XhoI restriction site sat in the sequence TAACTC GAGTAAAAGCTT in such a way that after the Fc-fragment restriction a new termination codon appeared. pShuttle-cPA was retrieved from the pShuttle-CMV-sPA plasmid by deleting the site containing the leader sequence peptide tpa using the NotI and NdeI restriction endonucleases.

The method of homologous recombination was used to generate the replication-defective adenoviruses Ad-PA-Fc, Ad-cPA, Ad-sPA. For this purpose, the plasmids pShuttle-CMV-PA-Fc, pShuttle-CMVcPA, and pShuttle-CMV-sPA were linearized by PmeI, mixed with the pAd-Easy (Adenoviral vector system, Stratogen), and then cotransformated into *E. coli *(BJ5183 strain). The obtained recombinant clones were used to extract plasmid DNA, whose molecular weight was later assessed. The *E. coli *of the DH5alpha strain were transformed with plasmids larger than 20 kbp due to the fact that this strain, unlike BJ5183, allows one to produce preparative amounts of the recombinant plasmids. The purified plasmid clones were analyzed both by splitting with the HindIII restriction endonuclease and PCR .

At the next step, we studied the infectivity of the described plasmids to permissive cells. Cells of the 293 line were transfected with the plasmids pAd-PA-Fc, pAd-sPA, and pAd-cPA, all linearized by PacI-sites. Transfection was performed in a 24-well plate using Lipofectamine 2000 (Invitrogen). Ten days after the transfection, the cells were collected and subjected to a freeze-thaw cycle; the obtained lysate containing recombinant adenoviruses was used to infect 293 cells in a 35-mm dish. After 5 days, specific lysis caused by the cytopathic effect of the recombinant viruses was detected. The lysate was used to extract DNA and perform an analysiss with PCR . The cell lysate was demonstrated to contain DNA of the recombinant human adenovirus serotype 5 carrying insertions that encode the protective antigen.

**Virus accumulation**

To accumulate recombinant adenoviruses serotype 5, we used a culture of 293 cells. A cell monolayer with a confluence of 50–70% was infected with the lysate of 293 cells, which, in turn, had been previously infected with recombinant Ad with a concentration of 10^7^ PFU per 15-cm plate. After 48 h, we collected the infected cells, concentrated them by low-speed centrifugation, suspended them in a buffer (0.01 M TrisHCl pH 8.0, 0.01 M NaCl, 5 mM EDTA), and disrupted them by triple freezing-thawing. The obtained suspension was centrifuged at 2000 rpm over 10 min at +4°C; the pellet was removed. The supernatant recombinant adenoviruses were purified via cesium chloride equilibrium density gradient centrifugation. As a result, we obtained 3 recombinant human adenoviruses serotype 5 carrying different insertions: Ad-cPA, Ad-sPA, and Ad-PA-Fc. Dilution of the recombinant adenovirus product was estimated by means of the plaque-forming method using HEK-293 cells.

**Expression of the recombinant proteinsbased adenovirus vector**

Expression of three recombinant proteins containing the *B. anthracis *protective antigen was analyzed by Western blotting with monoclonal antibodies. A specimen of the protective antigen purchased from Calbiochem (176908-100UG) was used as a positive control. A549 cells were transduced with recombinant adenoviruses; after 48 h, expression of the protective antigen in the supernatant and lysed cell pellet was assessed. To analyze the Fc-domain of the antibody within the fusion protein, we resorted to Western blotting with anti-species antibodies against mice IgG, conjugated to horseradish peroxidase (Amersham).

**Production of the protective antigen in *E. coli***

A plasmid carrying the gene of the receptor domain was designed with the use of the commercial vectors pUC 19 and pET 28b (Novagen). We used total DNA extracted from the *B. anthracis *strain 71/1 as a matrix. The cloning was performed with NdeI and E coRI as restriction sites. The forward and the reverse primers used for PAGR4 were GAGATC ATATGGTT GGGGCGGATGAG and ATCTC GAATTCTT ATCCT ATCTC ATAGCC , respectively. PCR fragments were extracted using kits (GE, Inc.) in accordance with their specifications. Fragments obtained after the restriction by NdeI and E coRI sites (Fermentas) were subcloned into the pUC 19 vector (*E. coli *JM109 was a recipient), and then into the pET 28b vector. To extract the recombinant proteins, *E. coli *BL21 were transformed with the designed vectors. The bacterial culture grew first in a LB medium until *OD*_600_ = 0.6–0.8, then for 3.5–4 h with IPTG (Sigma) at a concentration of 10 mM. After that, we centrifuged the bacterial mass for 15 min at 8000 *g* and then re-suspended the bacterial pellet in PBS. Subsequently, we sonicated the cells (in 3 steps, 30 s each, by an MSE disintegrator (England)) and extracted inclusion bodies by centrifugation for 40 min at 20000 *g*. The obtained pellet of inclusion bodies was dissolved in a 8-M urea solution; recombinant proteins were extracted according to a specification to N i-NT A-sepharose (Invitrogen), using thrombin (Sigma).

**Immunogenicity**

Immunogenicity of the recombinant adenoviruses encoding *B. anthracis *antigens was assessed in mice. Balb/c mice were immunized with recombinant adenoviruses twice within an interval of 2 weeks. A native protective antigen fused to an incomplete Freund’s adjuvant was employed as the positive control and administered by subcutaneous injection. A recombinant adenovirus without the antigen (Ad-null) served as the negative control. Recombinant adenoviruses were administered intranasally at a dose of 4.6 × 10^9^ PFU/ mouse and in a volume of 100 μl. Two weeks following the second immunization, we drew blood samples, extracted serum, and performed its examination for antibodies.

**Immunization**

The recombinant adenoviruses were introduced into the experimental animals intranasally at a dose of 15 × 10^9^ PFU/mice. The protective antigen with the Freund’s adjuvant was injected subcutaneously at a dose of 8–10 µg.

**Experimental animals**

We used female Balb/c mice with a weight of 20 g.

**Challenge**

To assess the protective ability of the potential genetic vaccine, the immunized animals were infected by means of intraperitoneal inoculation of the test culture at a dose of 4 LD_50_. An acapsular strain *Sterne B. anthracis *was employed as an infectious agent. Both the experimental and control groups of animals were monitored for 10 days following the infection. All experiments involving animals were carried out at the National Research Institute for Veterinary Virology and Microbiology of Russia, Russian Academy of Agricultural Sciences (RAAS).

**Statistical analysis**

The statistical analysis of the results was performed in Statistica 6.0. The results of the comparison of the experimental and the control group were considered statistically reliable at *p* < 0.05. The survival rate was evaluated using the Mann–Whitney U-test.

## RESULTS AND DISCUSSION

**Design of the recombinant adenoviruses**

**Fig. 1:**
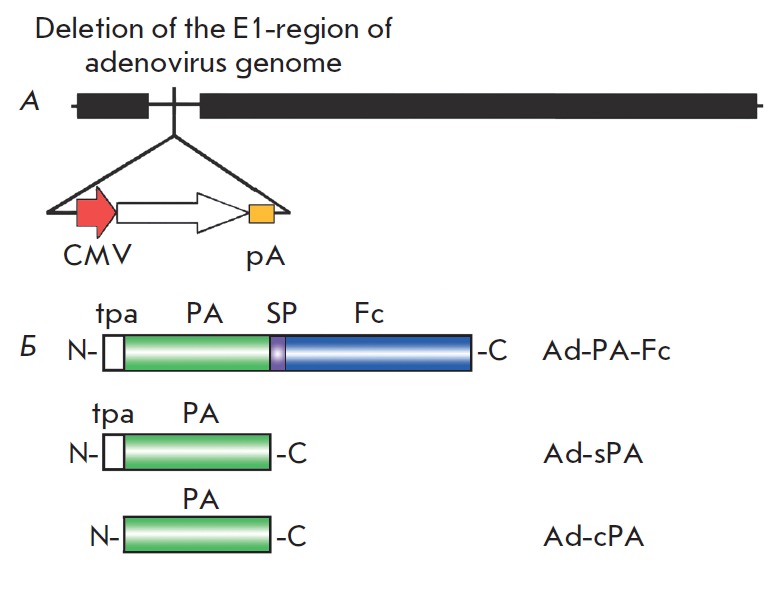
Layout of the recombinant adenovirus genome carrying the protective antigen of *B. anthracis. A *– human recombinant adenovirus serotype 5 genome. Expression cassette is inserted into where the E1 region was deleted. CMV – cytomegalovirus promoter; pA – polyadenylation signal. *B *– antigen schematic structure; tpa – tissue plasminogen activator, PA – protective antigen, SP – glycine serine spacer, Fc –Fc-fragment of the IgG2a antibody

The amino acid and nucleotide sequences of the fourth domain of the PA antigen were derived from U ni- ProtKB/Swiss-Prot P13423 and GenBank M22589.1. Analysis of the PA codons of *B. anthracis *showed that many codons did not suit well for expression in eukaryotic cells. The most frequent codons of *B. anthracis* and *M. musculus *were defined according to the codon usage database http://www.kazusa.or.jp/codon/. In order to achieve a high rate of protein production, the codons were optimized to be translatable in the *M. musculus* cells. The amino acids and nucleotide sequences of the Fc-fragment of IgG2a were obtained from U ni- ProtKB/Swiss-Prot P01863 and GenBank V00798.1. A 12-membered glycine-serine spacer was inserted between the PA antigen and Fc-fragment of the antibody (*Fig. 1*). Three shuttle vectors were designed on the basis of one pShuttle-CMV-PA-Fc plasmid with a number of fragments consecutively deleted.

**Properties of *in vitro *synthesized Ad-cPA, Ad-sPA, and Ad-PA-Fc**

**Fig. 2:**
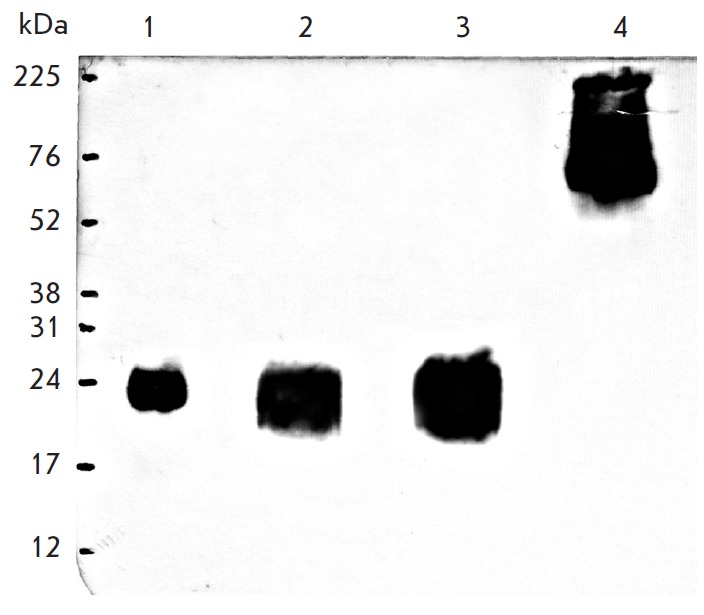
Results of PA detection carried out by means of Western blotting. 1 – purified fourth domain of the receptor antigen produced in *E. coli*; 2 – lysate of the cells infected with Ad-cPA; 3 – supernatant of the cells transduced with Ad-sPA; 4 – supernatant of the cells transduced with Ad-PA-Fc

In order to obtain data on the expression and secretion of PA as part of a recombinant adenovirus, A549 cells were retransduced with three adenovirus constructs. The fourth domain of PA, produced in E. coli, was employed as the positive control. After 48 h of incubation, we assessed the presence of the fourth domain of PA in both the supernatant and pellet left after the infected cells (*Fig. 2*). The supernatant of the cells transduced with the recombinant adenoviruses Ad-sPA and Ad- PA-Fc (*lanes 3*, *4*), as well as the lysed cells transduced with the recombinant adenovirus Ad-cPA (*lane 2*), contained the mentioned domain. The purified PA fourth domain synthesized in *E. coli *also showed a positive result (*lane 1*).

**Immune response induced by the recombinant adenoviruses Ad-cPA, Ad-sPA, and Ad-PA-Fc *in vivo***

**Fig. 3:**
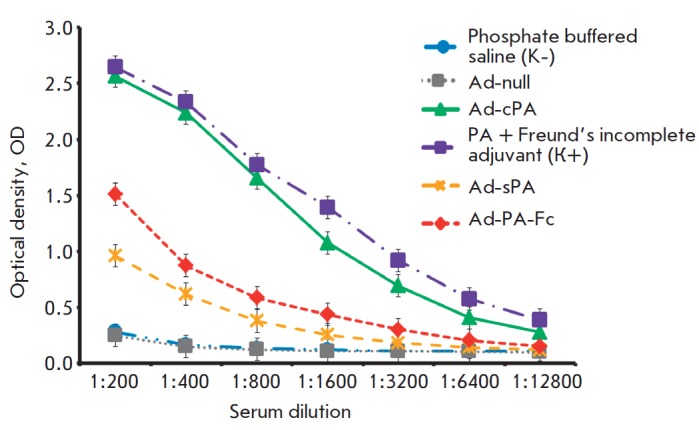
Results of anti-PA antibodies in the blood serum of mice immunized with recombinant adenoviruses by ELISA. Ad-PA-Fc – recombinant adenovirus containing the PA fused to the Fc-fragment of IgG2a; Ad- cPA – recombinant adenovirus containing a non-secretable form of PA; Ad-sPA – recombinant adenovirus containing the secretable form of PA. Positive control – the PA protein fused to an incomplete Freund’s adjuvant. Negative control – phosphate buffered saline (PBS)

The immune response to the PA fourth domain expressed as part of the recombinant adenoviruses was studied on Balb/c mice. The mice were immunized twice within an interval of 2 weeks. Ten days following the second immunization, mouse blood was examined for specific antibodies against the PA in the serum by means of ELISA (*Fig. 3*). An unexpected result was that the blood serum of mice infected with Ad-cPA revealed a high level of specific antibodies similar to that observed in the mice immunized with the PA fused to the incomplete Freund’s adjuvant. The blood serum of mice infected with Ad-sPA and Ad-PA-Fc demonstrated almost the same concentration of specific PA antibodies, which was lower than that for Ad-cPA.

**Defining subclasses of the specific IgG**

**Fig. 4:**
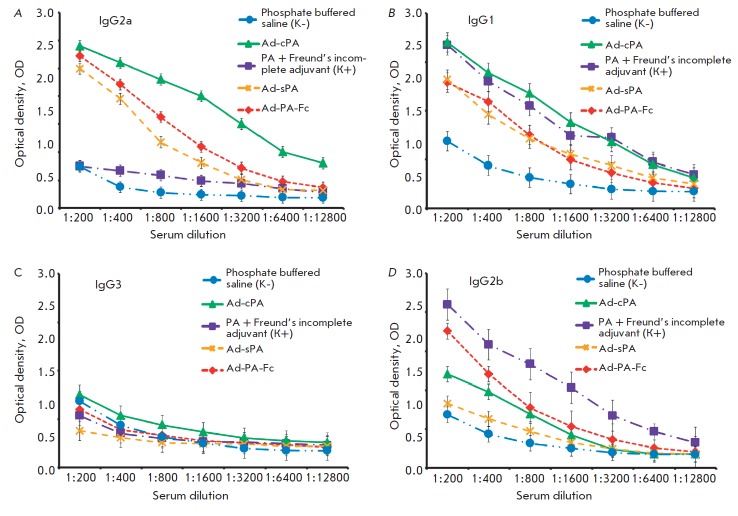
Results of detection of the specific IgG against the protective antigen by ELISA in the blood serum of mice immunized with Ad-cPA, Ad-sPA, and Ad-Fc-PA. A – amount of IgG2a; B – IgG1; C – IgG3; D – IgG2b. Ad-PA-Fc – recombinant adenovirus containing the protective antigen fused to the Fc-fragment of IgG2a; Ad-cPA – recombinant adenovirus containing a non-secretable form of PA; Ad-sPA – recombinant adenovirus containing a secretable form of PA. Positive control – PA protein fused to a incomplete Freund’s adjuvant. Negative control – phosphate buffered saline (PBS)

The sera obtained at the previous step were also tested for subclasses of the specific IgG to the PA-antigen (*Fig. 4*). It was demonstrated that all recombinant adenoviruses induced a high-level production of IgG2a and IgG1 (*Fig. 4 A, B).* The blood serum of the mice immunized with Ad-cPA and Ad-Fc-PA contained antibodies of the IgG2b subclass (*Fig. 4D*). None of the mice groups, including that of the positive control, revealed IgG3 in their blood serum (*Fig. 4C*). It is inter esting to mention that immunization with the PA fused to the incomplete Freund’s adjuvant failed to induce the production of IgG2a, but it triggered that of IgG2b and IgG1 subclasses.

**Examination of the protective abilities of the recombinant adenoviruses by the control challenge**

**Table 1:** Protective ability of genetic vaccines based on the recombinant adenoviruses in Balb/c inbred white mice as a model

Group of animals	Immunizing product	Balb/c mice	Survived	Died	Protection, %
1	PA + IFA	10	3	7	30
2	Ad-PA-Fc	10	9	1	90
3	Ad-cPA	10	8	2	80
4	Ad-sPA	10	8	2	80
5	Ad-null	10	2	8	20

To assess the protective ability of the humoral immune response induced by the designed recombinant adenoviruses, we infected the immunized mice with a lethal dose of the *B. anthracis, Sterne *strain (4 LD_50_). In a week, 80% of the control group animals vaccinated with the recombinant adenovirus without insertion (Adnull) died. All the constructs with the protective antigen provided protection against *B. anthracis *in 80–90% of the animals (*Table 1*).

**Fig. 5:**
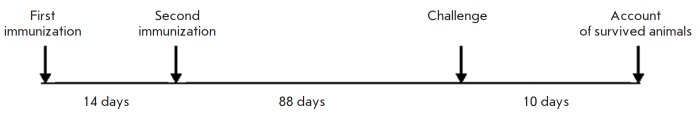
Schematic view of the protection of immunized animals against *B. anthracis. *Recombinant adenoviruses were administered intranasally at a dose of 4.6 × 10^9^ PFU/mouse, in a volume of 100 μl. Native PA protein with an incomplete Freund’s adjuvant was employed as a positive control

The duration of a strong immunity against anthrax was preliminary estimated as follows (*Fig. 5*). Balb/c mice were immunized with the recombinant adenoviruses at a dose of 4.6 × 10^9^ PFU/mouse within an interval of two weeks. Eighty-eight days later, they were infected with *B. anthracis *of the *Sterne *strain (4 LD_50_).*Table 2*shows that after the introduction of *B. anthracis* of the *Sterne *strain, 90–100% of the mice vaccinated with the recombinant adenoviruses survived. Mice immunized with Ad-PA-Fc demonstrated a stronger immunity than those immunized with Ad-sPA and Ad-cPA. Moreover, animals immunized with Ad-sPA and Ad-cPA lost weight and showed signs of sickness, unlike those immunized with Ad-PA-Fc.

## DISCUSSION

In 2003 Y. Tan designed the first recombinant adenovirus serotype 5 encoding a protective antigen modified to be expressed in human cells [14]. It was demonstrated that intramuscular introduction of 10^9^ viral particles induced the production of anti-PA-antibodies in an amount 2.7 times higher than that produced upon immunization with the subunit human vaccine used in the USA. It is remarkable that the recombinant adenovirus induced a faster humoral immune response than the subunit vaccine. When immunization was followed by administration of the anthrax toxin, mice immunized with the recombinant adenovirus demonstrated immunity in 75% of cases, whereas the subunit vaccine provided protection only in 25% of cases.

In 2005, the same authors performed an analogous study for the recombinant adenovirus serotype 7 [15]. They demonstrated that the preexisting immune response to adenovirus serotype 5 in mice can be overridden by an adenovirus of a different type and that this allows one to protect animals from a lethal dose of the anthrax toxin.

A similar feature is that not the entire amino acid sequence of the PA was employed as an antigen, but only its fourth domain. The reason behind this was that the mentioned domain was essential for binding the cell receptor: so, one could achieve a protective immune response by blocking the domain with antibodies. For example, Z. Yu *et al*. designed two plasmids carrying codes of secretable and non-secretable forms of the PA fourth domain [16]. After the immunization of animals with these plasmids, an increase in the IFN-γ level was observed along with the secretion of anti-PA- antibodies. The authors of another study, J. McConnell* et al*. [13], achieved expression of the PA fourth domain encoded by the adenoviral vector. At a single immunization with subsequent introduction of a lethal dose of the anthrax toxin, experimental animals demonstrated immune protection in 67% of cases. The next study by the same authors was devoted to the protective effect of the recombinant adenovirus in mice infected with a lethal dose of the vaccine strain 34F2 [17]. They resorted to prime-boost immunization. Priming with plasmid DNA followed by boosting with the recombinant adenovirus, as well as priming with recombinant adenovirus followed by boosting with the recombinant adenovirus, fully protected mice against *B. anthracis*. These results indicate that vaccination with the recombinant adenovirus protects against anthrax infection, and that this approach can be effective in immunization against bacterial and viral pathogens.

The remarkable feature of our study was that we demonstrated that dimerization of the protective antigen with the Fc-fragment of the antibody increases the immunogenicity of the former and the ability of the above-mentioned fragment to bind specifically to macrophages through Fc-receptors and activate the complement system via the classical pathway.

The dimer-forming ability allows two antigen determinants to reside in the same particle, which increases the immunogenicity of the fusion protein [18, 19]. Activation of multichain immune recognition receptors (MIRR receptors) is probably a mechanism inducing the increase in immunogenicity. When ligands bind, MIRR receptors transduce the signal via ITAMs, whose activation leads to the merging of immune complexes and to the merging of endosomes with MHCII-containing vesicles [20, 21].

On the other hand, if the unit of the fusion protein capable of oligomerization or the antigen itself can interact with the pattern recognition receptor, this helps increase immunogenicity up to a maximum and to avoid additional adjuvants [18]. As a result, the protein capable of oligomerzation acts as a “molecular adjuvant.” In our study, we added the Fc-fragment of IgG2a of *M. musculus *to the protective antigen in the fusion protein. The Fc-fragment of the antibody can activate the classical pathway of the complement system, whose pattern recognition receptors (PR) deliver an essential co-stimulatory signal [22]. The combined actions of MIRR and PR receptors in the same cell result in the presentation of the antigen peptides to helper T lymphocytes, which can assist both T- and B-cells. Possibly, this is the reason as to why the above-mentioned recombinant adenovirus carrying PA-Fc provides a stronger protection than Ad-sPA and Ad-cPA (see *Table 1*).

Another aspect that is important to mention is the use of an adenovirus vector as a carrier of the synthesized gene. Due to the fact that the gene is synthesized inside the cells transduced with the recombinant adenovirus, some antigen molecules undergo processing and peptide presentation, together with MHC I molecules. The complexes that are formed on activated cells induce cytotoxic T-lyphocytes, which aid in the protection against intracellular pathogens, including* B. anthracis* [14, 15, 23]. In our study, in order to estimate the activity of cytotoxic T-lymphocytes, we designed the recombinant adenovirus Ad-cPA carrying a non-secretable modification of the protective antigen and showed it to protect 89% of experimental animals (*Table 2*). On the other hand, the secretable form of the protective antigen (Ad-sPA) provided almost the same level of protection without “molecular adjuvants.” Thus, the results we obtained fully correlate with those obtained by other groups of researchers [16]. We assume that the use of the recombinant adenoviral vectors Ad-sPA and Ad-cPA helps to induce antigenspecific cytotoxic T-lymphocytes.

**Table 2:** Protective ability of genetic vaccines based on the recombinant adenoviruses in Balb/c inbred white mice as a model

Group of animals	Immunizing product	Balb/c mice	Survived	Died	Protection, %
1	Ad-PA-Fc	9	9	0	100
2	PA + IFA	9	9	0	100
3	Ad-sPA	9	8	1	89
4	Ad-cPA	9	8	1	89
5	Ad-null	9	1	8	11.1
6	Ad-null	9	1	8	11.1

In the present study we resorted to the intranasal introduction of adenoviral vectors, because it is this particular way of delivering recombinant adenoviruses that helps to override the preexisting immune response to the vector [24]. Intranasal introduction has a number of advantages in comparison with other ways: it is a needle-free, non-invasive and painless procedure that requires no medical staff and can be performed by the vaccinated individuals themselves. In addition, the data obtained by J. Zhang *et al*. [24] demonstrated that a single intranasal immunization with a recombinant adenoviral vector carrying the gene of the protective antigen protects mice against* B. anthracis *on condition of preexisting immune response to the adenoviral vector.

It is interesting to note that immunization with recombinant adenoviruses induces IgG to belong to the same class as the protective antigen. Our experiments revealed antibodies belonging to the subclasses that are produced at an immune response of the Th1 type (characterized by the highest level of IgG2a antibodies), whereas when an incomplete Freund’s adjuvant is used, an immune response of the Th2 type (characterized by the highest level of IgG2b antibodies) is induced. One may assume that this was the reason as to why mice immunized with the recombinant adenoviruses during the first experiment were better protected than those from the positive control group (*Fig. 4*) (*Table 1*). Our results correlate with the data of Y. Tan* et al. *[14], who demonstrated an analogous profile of the antibody subclasses upon adenoviral immunization. A remarkable distinction of our study was the total absence of IgG3, whereas Y. Tan’s group detected the above-mentioned subclass, even though in a lesser amount than that of other IgG.

It is remarkable that there can be two classes of antibodies produced in response to the *B. anthracis *protective antigen. The first type of antibodies are able to bind single molecules of the protective antigen and neutralize them, i.e., to sterically block protein-protein interactions between the PA molecules. Antibodies of the second type interact only with the oligomeric complexes of the protective antigen and change their structure in such a way that those are no longer able to interact with the receptors on the cell surface [25]. This means that antibodies of the second type are produced only in the presence of oligomerized molecules of the antigen, a complex of which threads through the membranes of eukaryotic cells. Since the fourth domain of PA resides on the C-end of the protein, and in our construct (PA-Fc) it resides on the N -end (to provide the Fc-fragment of the antibody with a higher functional activity), we designed a recombinant adenovirus carrying the Fc-fragment at the N -end of the protein and the fourth domain of PA – at the C-end of the protein. The designed recombinant adenovirus (Ad-Fc-PA) was studied in the described experiments. It was shown that it exhibited the same immunogenic and protective properties as the recombinant adenovirus Ad-PA-Fc (data are not presented).

## CONCLUSIONS

We have shown that immunization with the recombinant adenovirus protects against the acapsular strain of *B. anthracis. *Ad-PA-Fc carrying the Fc-fragment of the antibody fused to the protective antigen provides a higher level of protection compared to Ad-sPA and Ad-cPA. Besides, immunization with Ad-PA-Fc can confer full immunity to mice during the following three months. Our plans for the future are to experiment on guinea pigs with sporules of endospore-forming strains of *B. anthracis*.

## Abbreviations

Ad: adenovirus
PFU: plaque-forming unit
PA: protective antigen
Fc: Fc-fragment of IgG2a
IFA: incomplete Freund’s adjuvant
PBS: phosphate-buffered saline
